# Self‐Coacervation of Oligo(ethylene glycol) and Oligo(2‐ethyl‐2‐oxazoline)‐Based Double Hydrophilic Brush Block Copolymers in Aqueous Solution

**DOI:** 10.1002/marc.202500151

**Published:** 2025-04-18

**Authors:** Niamh Bayliss, Matilde Concilio, Alexander Plucinski, Gokhan Yilmaz, C. Remzi Becer, Bernhard V. K. J. Schmidt

**Affiliations:** ^1^ School of Chemistry University of Glasgow Glasgow G12 8QQ UK; ^2^ Department of Chemistry University of Warwick Coventry CV4 7AL UK

**Keywords:** aqueous multi‐phase system, coacervation, double hydrophilic block copolymer, poly(2‐oxazoline), self‐assembly

## Abstract

Coacervates are a highly relevant class of structures formed via liquid–liquid phase separation and new coacervate‐forming polymers are highly sought after. Here, the formation of simple coacervate droplets from a double hydrophilic block copolymer (DHBC) with the combination of poly(oligo ethylene glycol methacrylate) (POEGMA) and poly(oligo 2‐ethyl‐2‐oxazoline methacrylate) (POEtOx) without the use of external triggers or charges is shown. At a high concentration of 25 wt.%, the DHBC forms coacervate droplets with sizes in the low micrometre range. The droplets have relatively high stability over a long period of time with only minor coalescence observed after 4 weeks. At low concentrations, no coacervation is observed. Furthermore, copolymers of the monomers also do not show coacervation clearly indicating that the DHBC architecture is required to form the desired structures. The addition of guest polymers and biomacromolecules at low concentrations shows a specific partitioning behaviour with a preference for the polymer or the aqueous phase. At high guest concentrations, enrichment in the aqueous phase is observed, in line with common water‐in‐water (w/w) emulsions. These findings constitute a new direction for coacervate systems that are of interest for biotechnology, synthetic cell environment mimics and drug delivery.

## Graphical abstract

Here, the formation of double hydrophilic brush block copolymer‐based simple coacervates is presented. The system phase separates into polymer‐enriched droplets at elevated concentrations. The addition of guest polymers and biomacromolecules shows a distinct partitioning behaviour. At elevated guest concentrations, a stable phase system similar to water‐in‐water emulsions is observed even though no stabilizer is required.

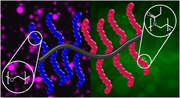

## 1 Introduction

Hydrophilic polymers have a wide range of applications, such as wastewater treatment,^[^ ^1^, ^2^ ^]^ drug delivery^[^ ^3^, ^4^ ^]^ and biomolecule purification.^[^ ^5^, ^6^, ^7^ ^]^ The scope of these applications continues to expand, with recent interest in replicating crowded cellular environments using hydrophilic polymers.^[^ ^8^, ^9^ ^]^ In particular, coacervates have gained attention as models for cellular environment mimics. They are formed through liquid–liquid phase separation (LLPS) of polymers, resulting in a polymer‐rich and a polymer‐depleted phase. There are two different types of coacervation, simple coacervation and complex coacervation.^[^ ^10^ ^]^ Coacervates formed from a single polymer only are known as simple coacervates.^[^ ^11^ ^]^ For example, simple coacervates formed from poly(acrylic acid) stabilized with agar have been used as an environment for enzymatic catalysis, featuring an all aqueous system required for preserving enzyme activity.^[^ ^12^ ^]^ Complex coacervation involves two or more polymeric components and typically occurs due to electrostatic interactions between the polymeric species. This process employing polypetides has previously been used to mimic LLPS into membrane‐less condensates present in cellular environments and to help understand the ways in which biological systems function.^[^ ^13^ ^]^ Our group showed an example of associative LLPS leading to complex coacervates from two different polyacrylamides without the use of charge interactions.^[^ ^14^ ^]^ Mann and coworkers studied the production of nitric oxide in a coacervate environment via cascade catalysis.^[^ ^15^ ^]^ Thus, the use of hydrophilic polymers to form both complex and simple coacervates results in all aqueous biocompatible microreactors.^[^ ^16^, ^17^ ^]^ Coacervate formation is typically conducted through external triggers such as additional charges, temperature or pH changes. Being able to form polymer coacervates in the absence of external triggers can potentially bring a wider range of uses in biocompatible microreactors.^[^ ^18^, ^19^ ^]^

Another method to form aqueous multi‐compartment systems is through aqueous two‐phase systems (ATPSs), which require high polymer concentrations to achieve phase separation.^[^ ^20^ ^]^ ATPSs generally occur when two immiscible polymers are dissolved in water, resulting in the formation of two phases, one enriched in one polymer and the other enriched in the other polymer. Since both polymers are hydrophilic, the interfacial tension between the phases is very low compared to oil‐in‐water systems, making ATPS ideal for mimicking bio‐condensates in cellular environments. The aqueous environment of ATPSs also makes them ideal for the purification and separation of biomolecules.^[^ ^7^, ^21^ ^]^

These ATPSs can be further used to form water‐in‐water (w/w) emulsions, without the need for organic solvents, improving biocompatibility.^[^ ^22^ ^]^ Stabilization can be achieved using particles to form Pickering emulsions, such as inorganic nanoparticles,^[^ ^23^ ^]^ polymer nanoparticles,^[^ ^24^, ^25^ ^]^ microgels^[^ ^26^, ^27^ ^]^ or polymer micelles.^[^ ^28^, ^29^ ^]^ The macroscopic phase separation observed with homopolymers in ATPSs can be translated into microscopic phase separation when double hydrophilic block copolymers (DHBCs) are used. In contrast to the self‐assembly of amphiphilic block copolymers driven by hydrophobic interactions, DHBC self‐assembly is purely hydrophilic. The difference in hydrophilicity between the blocks causes demixing due to varying osmotic pressures, leading to self‐assemblies such as vesicular aggregates.^[^ ^30^ ^]^ The formation of DHBC‐based aggregation is highly dependent on concentration. At low concentrations (usually below 10 wt.%) colloidal structures, such as micelles, vesicular aggregates and particles, have been observed.^[^ ^30^, ^31^, ^32^, ^33^ ^]^ At elevated concentrations (above 20 wt.%), DHBC self‐assembly results in the formation of polymer‐rich droplets surrounded by a polymer‐depleted aqueous phase or vice versa. The formation of a polymer‐concentrated‐coacervate is driven by hydrogen bonding between the two separate blocks.^[^ ^34^, ^35^ ^]^ Further increase in concentration leads to the formation of continuous assemblies like lamellar or hexagonal domains.^[^ ^36^, ^37^, ^38^ ^]^ As the entire system is hydrophilic, unlike amphiphilic self‐assemblies, the DHBC self‐assemblies exhibit a high permeability and compatibility for biotechnological and biomedical applications.^[^ ^39^ ^]^

**Scheme 1:**
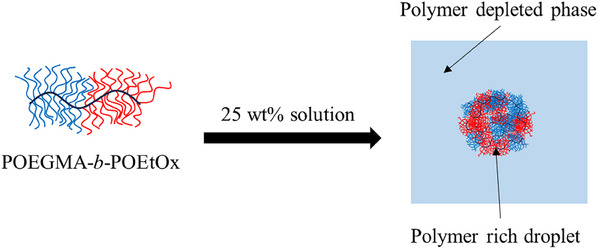
Representation of POEGMA‐*b*‐POEtOx block copolymer and coacervates formed at 25 wt.% aqueous solutions.

Herein, we explore the use of DHBC to form coacervates using a novel block copolymer consisting of a poly(oligo ethylene glycol methacrylate) segment and a poly(oligo 2‐ethyl‐2‐oxazoline methacrylate) segment (POEGMA‐*b*‐POEtOx) (**Scheme** **1**). Specifically, we report the formation of simple coacervates of POEGMA‐*b*‐POEtOx formed without external triggers, where we examine the effects of molecular weight, concentration, and conformation of the polymers on coacervation. Additionally, we investigate the distribution of various biomacromolecules using confocal laser scanning microscopy (CLSM), exploring the potential applications of the coacervates in purification, drug delivery, or as bio‐microreactors.

## 2 Results and Discussion

### 2.1 Block Copolymer and Copolymer Synthesis

**Scheme 2:**
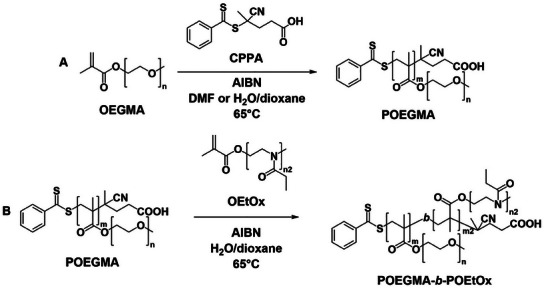
POEGMA‐*b*‐POEtOx block copolymer synthesis. A) Initial OEGMA polymerization. B) OEtOx block polymerization with the initial POEGMA block as the macro chain transfer agent.

The DHBC were obtained by combining OEGMA and OEtOx macromonomers (**Scheme** **2**).^[^ ^40^ ^]^ For this purpose, the macromonomers containing EtOx with a molecular weight of 1000 g mol^−1^ were synthesized, while commercial OEGMA was employed.

The OEtOx macromonomer was synthesized via cationic ring‐opening polymerization (CROP) of EtOx, followed by end‐capping with methacrylic acid (MAA). The CROP of EtOx was performed in acetonitrile (CH_3_CN) at 100 °C using methyl tosylate (MeTos) as the initiator, with a final monomer concentration of 4.0 m, and a [monomer]:[MeTos] ratio of 10:1. Upon reaching full conversion, the living oxazoline chains were end‐capped by directly adding a CH_3_CN solution containing a five‐fold excess of MAA and *N*,*N*‐diisopropylethylamine (DIPEA). The reversible addition‐fragmentation chain transfer (RAFT) polymerization of OEGMA was carried out in DMF or a 1:4 mixture of deionized water and dioxane at 65 °C using 4‐cyano‐4‐(phenylcarbonothioylthio)pentanoic acid (CPPA) as the chain transfer agent (CTA), and azobisisobutyronitrile (AIBN) as the initiator. The final monomer concentration was maintained at 0.33 m, while the [OEGMA]:[CTA]:[AIBN] ratio was adjusted based on the desired molecular weight. This way, three macro chain transfer agents were synthesised with molecular weights of 4000 g mol^−1^ (POEGMA_4k_), 9000 g mol^−1^ (POEGMA_9k_), and 13000 g mol^−1^ (POEGMA_13k_)_._ Because of the presence of the CTA in these initial blocks, further polymerization with the OEtOx macromonomer was conducted to produce three different block copolymer samples (POEGMA‐*b*‐POEtOx), targeting a 1:1 molar ratio between the individual blocks. Due to issues with the reinitiation of the macro‐RAFT agent, a 1:4 mixture of deionized water and dioxane was used. The DHBCs were further labelled with Rhodamine isothiocyanate (RITC) to enable CLSM imaging. Control polymers were also synthesized by copolymerizing OEGMA and OEtOx macromonomers (P(OEGMA‐*co*‐OEtOx)). The macromonomer ratios were varied to obtain copolymers with the same overall composition as the DHBCs. These polymers were labelled with fluorescein isothiocyanate (FITC) for CLSM imaging. FTIC was chosen to produce labelling to be distinguishable from the DHBCs.

**Figure 1:**
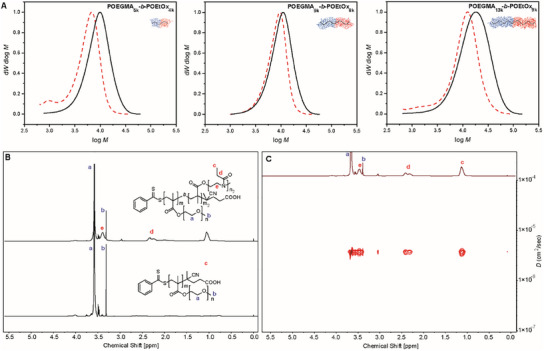
A) GPC‐derived molecular weight distributions obtained in 0.1 m NaNO₃ (aq.) and MeOH (80:20% v/v) with PEG calibration, wherein initial POEGMA macro chain transfer agents are shown in dashed red and block copolymers shown in black; POEGMA_5k_‐*b*‐POEtOx_4k_ (left), POEGMA_9k_‐*b*‐POEtOx_8k_ (middle) and POEGMA_13k_‐*b*‐POEtOx_9k_ (right). B) ^1^H NMR of POEGMA_5k_ (bottom) and POEGMA_5k_‐*b*‐POEtOx_4k_ (top). C) DOSY of POEGMA_5k_‐*b*‐POEtOx_4k_ in CDCl_3_.

Block copolymer formation was confirmed using gel permeation chromatography (GPC), ^1^H NMR, and diffusion‐ordered spectroscopy (DOSY). Both the POEGMA macro chain transfer agents and the DHBCs exhibited unimodal molecular weight distributions with low dispersities (*Ð* < 1.7) (**Figure** **1**A). Additionally, chain extension of the POEGMA macro‐RAFT agent with OEtOx resulted in a molecular weight shift to higher values, confirming successful polymerization. In contrast, the OEGMA/OEtOx copolymers exhibited slightly bimodal molecular weight distributions, probably due to the interaction of the polymers with the GPC column (Figure S1, Supporting Information).

Apparent molecular weights were determined by GPC in 0.1 m NaNO₃ (aq.) and MeOH (80:20% v/v) according to PEG calibration (**Table** **1**). The obtained molecular weight values are lower than the theoretical ones due to the nature of brush polymers and the limitations of GPC analysis. The calculated molecular weights from calibration are not entirely accurate, as the hydrodynamic volume of brush polymers differs significantly from that of linear polymers, especially as the polymer length increases.

**Table 1:** Molecular weights and dispersity of POEGMA‐*b*‐POEtOx block copolymers, starting blocks and P(OEGMA‐*co*‐OEtOx) copolymers.

Polymer	*Conversion* [%]^a)^	*M_n,theo_* [g mol^−1^]^b)^	*M*_n,GPC_ [g mol^−1^]^c)^	*Đ* ^c)^	Mole Fraction POEGMA/POEtOx^d)^
POEGMA_5k_	64	4800	3700	1.61	–
POEGMA_9k_	89	8900	7200	1.23	–
POEGMA_13k_	67	12800	7000	1.82	–
POEGMA_5k_‐*b*‐POEtOx_4k_	76	8500	7300	1.41	0.52/0.48
POEGMA_9k_‐*b*‐POEtOx_8k_	80	16000	8600	1.32	0.51/0.49
POEGMA_13k_‐*b*‐POEtOx_9k_	77	22700	12300	1.66	0.60/0.40

^a)^ Determined from the ^1^H NMR spectra using the polymer peak at 3.30 ppm and the macromonomer peak at 6.0 ppm for POEGMA, the polymer peak at 1.0 ppm and the macromonomer peak at 6.1 ppm for POEtOx;

^b)^ Calculated from conversion;

^c)^ Obtained from GPC in 0.1 m NaNO₃ (aq.) and MeOH (80:20% v/v) calibrated with narrow PEG standards;

^d)^ Determined from the ^1^H NMR spectra of the block copolymers by comparing the terminal methyl signals for the POEGMA and POEtOx blocks around 3.30 and 2.95 ppm, respectively.

A comparison of the achieved molecular weights indicates that obtaining higher molecular weights is more challenging, likely due to the increased steric hindrance at the active chain ends during polymer growth. Given the discrepancy between the apparent molecular weight (${\bar{M}}_{n,\mathrm{GPC}\;}$) from GPC analysis and the theoretical molecular weight (${\bar{M}}_{n,\mathrm{theo}}$) calculated from the initial reagent ratios and final conversion, ${\bar{M}}_{n,\mathrm{theo}}$ is used for sample labelling.

^1^H NMR spectroscopy confirmed the formation of block copolymers as characteristic peaks for both blocks were observed (Figure 1B; Figures S2–S10, Supporting Information). The formation of block copolymer, rather than the mixture of two homopolymers, was further validated by DOSY, which showed a single distinct diffusion coefficient corresponding to the DHBC (Figure 1C; Figures S11–S13, Supporting Information). For the copolymers, signals for both macromonomers were present in the spectra, and the observed monomer ratios closely matched the feed composition, with only minor discrepancies.

### 2.2 Simple Coacervate Formation

**Figure 2:**
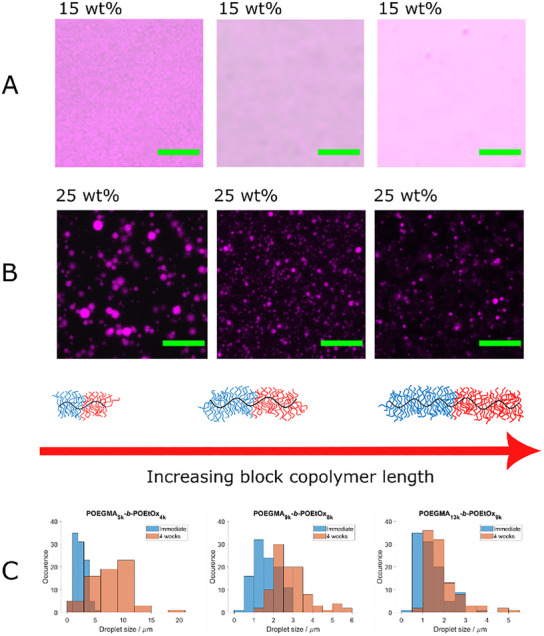
A) 15 wt.% POEGMA_5k_‐*b*‐POEtOx_4k,_ POEGMA_9k_‐*b‐*POEtOx_8k_ and POEGMA_13k_‐*b*‐POEtOx_9k_ in deionized water (1 wt.% of the respective RITC labelled POEGMA‐*b*‐POEtOx added) shown in magenta with varying molecular weight. B) 25 wt.% POEGMA_5k_‐*b*‐POEtOx_4k,_ POEGMA_9k_‐*b‐*POEtOx_8k_ and POEGMA_13k_‐*b*‐POEtOx_9k_ in deionized water (1 wt.% of the respective RITC labelled POEGMA‐*b*‐POEtOx added) shown in magenta with varying molecular weight. Block copolymer sizes increase from left to right. C) Coacervate droplet size distributions: POEGMA_5k_‐*b*‐POEtOx_4k_ (left), POEGMA_9k_‐*b‐*POEtOx_8k_ (middle) and POEGMA_13k_‐*b*‐POEtOx_9k_ (right). Green scale bar = 20 µm.

POEGMA‐*b*‐POEtOx block copolymer coacervates spontaneously formed in an aqueous solution at a concentration of 25 wt.% without the need for external triggers. In contrast to the self‐assembly of block copolymers, a LLPS separation occurs leading to the formation of droplets.^[^ ^10^ ^]^ To investigate the coacervate formation, 1 wt.% of the 25 wt.% block copolymer was RITC – labelled and imaged using CLSM. The analysis revealed polymer‐rich droplets surrounded by a continuous aqueous phase for all three block copolymers (**Figure** **2**). The coacervates formed were consistent in size, with POEGMA_5k_‐*b*‐POEtOx_4k_ exhibiting an average particle size of 2.50 ± 1.00 µm, POEGMA_9k_‐*b‐*POEtOx_8k_ measuring 1.64 ± 0.61 µm, and POEGMA_13k_‐*b‐*POEtOx_9k_ averaging 1.37 ± 0.66 µm (Figure 2; Table S1, Supporting Information). Notably, larger block copolymers lead to smaller droplets. The decrease in droplet size could be attributed to the more compact conformation of the longer block length which in turn causes the intermolecular interactions to decrease, decreasing the overall size and increasing the steric stability of the droplets at the same time. In the future, we will perform further studies to investigate the effects of block ratios on coacervate formation and stability.

The polymer solutions were re‐imaged using CLSM after a 4‐week period without agitation. Prior to imaging, the solutions remained as a single phase (Figure S14, Supporting Information), and CLSM confirmed the continued presence of coacervates (Figure S15, Supporting Information). Over time, the coacervates increased in size across all block copolymer samples: POEGMA_5k_‐*b*‐POEtOx_4k_ grew by a factor of 3.2 to an average size of 7.98 ± 3.45 µm, while POEGMA_9k_‐*b*‐POEtOx_8k_ and POEGMA_13k_‐*b*‐POEtOx_9k_ increased by a factor of 1.7 to 2.86 ± 0.92 µm and a factor of 1.4 to 1.90 ± 0.82 µm, respectively (Figure 2; Table S1, Supporting Information). This indicates a tendency for the coacervate droplets to coalesce over time, though the growth remains gradual. A clear trend in the coalescence was observed, where shorter DHBCs exhibited more pronounced droplet fusion, which might be attributed to the steric bulk of the polymers. Without additional stabilizers, droplet stability relies solely on steric stabilization, which is more pronounced in larger block copolymers.

Dilute solutions of the block copolymers did not exhibit coacervate formation. At a concentration of 15 wt.%, using 1 wt.% RITC‐labelled block copolymers, no coacervates were observed, indicating that a high polymer concentration is required for coacervate formation (Figure 2). We hypothesize that the attractive interactions of DHBCs are driven by hydrogen bonding between the blocks, leading to LLPS. To study the dependence of coacervate formation on DHBC architecture, the P(OEGMA‐*co*‐OEtOx) copolymers were also investigated. Aqueous solutions of the copolymers at a concentration of 25 wt.% showed no coacervate formation (Figure S16, Supporting Information). This result suggests that distinct blocks in the block copolymers are essential for coacervation. Evidently, microscopic phase separation is necessary to generate sufficient attractive interactions for coacervate formation. As these droplets consist of a single polymer component, they align with the definition of simple coacervates.^[^ ^10^ ^]^

### 2.3 Effect of the Addition of Biomacromolecules and Polymers on Coacervate Formation

**Figure 3:**
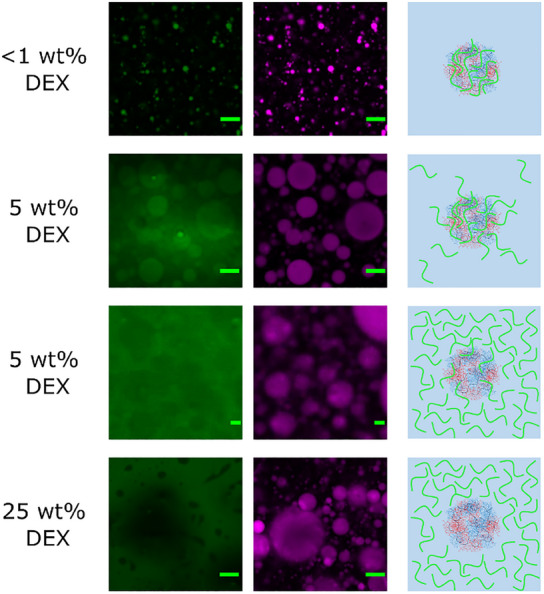
CLSM images of RITC‐labelled POEGMA_9k_‐*b‐*POEtOx_8k_ 25 wt.% aqueous solutions (magenta), along with FITC labelled DEX at increasing concentrations (green). Schematic depictions of the different phases, where green represents DEX and red and blue represent the polymer droplets. Green scale bar = 20 µm.

To investigate the effects of additional molecules on the coacervates formed by the block copolymers, FITC‐labelled dextran (DEX) was added to 25 wt.% POEGMA_9k_‐*b‐*POEtOx_8k_ aqueous solutions (**Figure** **3**). At DEX concentrations below 1 wt.%, DEX preferentially accumulated in the polymer‐rich phase, which was unexpected. According to the literature, DEX forms ATPSs with PEG and PEtOX,^[^ ^41^ ^]^ which suggests that DEX would partition into the polymer‐depleted phase. However, at low DEX concentrations, the attractive interactions between the polymers appear to outweigh any dissociative forces, favouring DEX accumulation in the polymer‐enriched coacervate phase. Higher DEX concentrations were also investigated. At 5 wt.% concentration, DEX exhibited mixed distribution: in some areas, it was present within the polymer droplets, while in others it was in the aqueous continuous phase. The separation at 5 wt.% was not as distinct as in the <1 wt.% sample and larger polymer droplets were observed, which might be due to the samples being shaken by hand for dispersion. The less well‐defined separation suggests that at 5 wt.%, DEX accumulates at the phase boundary and transitions between the polymer droplet phase and the aqueous phase. This shift compromises the steric stabilization of the coacervate droplets, resulting in coalescence. When the DEX concentration was further increased to match POEGMA_7k_‐*b‐*POEtOx_1k_ at 25 wt.%, DEX was found entirely in the opposite aqueous continuous phase. The separation of DEX to the aqueous continuous phase is presumed to be due to the increased volume fraction of DEX that overwhelms the coacervate phase and induces phase separation (Figure 3), which is a common effect for ATPS that depends strongly on the concentration of the constituting polymers and are preferred at elevated concentrations. The observed behaviour is similar to a w/w emulsion formed from ATPSs however, in this case, one of the phases is the coacervate phase. This result is in accordance with the literature on ATPSs, where the formation of w/w emulsions required stabilization. However, in the case of these simple coacervates, a w/w emulsion forms regardless of the addition of stabilizers.

**Figure 4:**
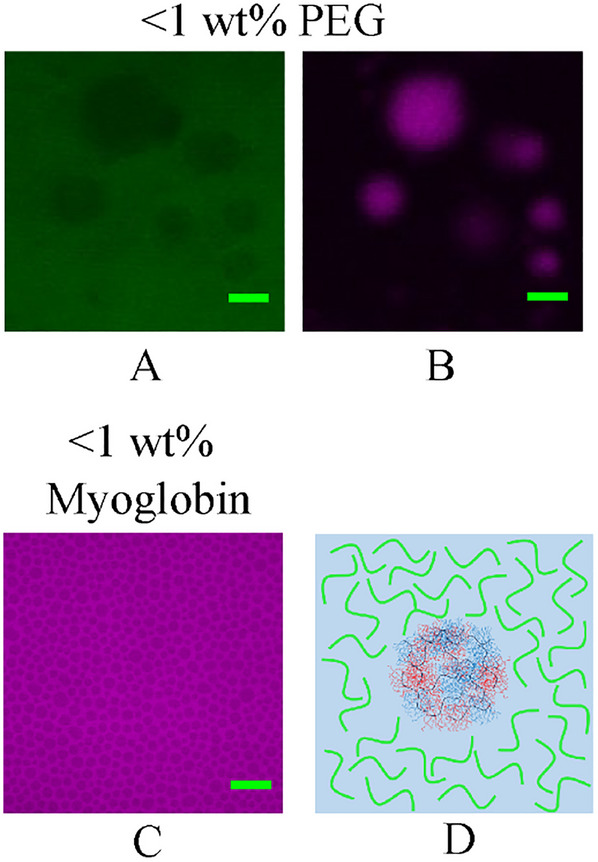
CLSM images of POEGMA_9k_‐*b‐*POEtOx_8k_ 25 wt.% aqueous solutions with A) <1 wt.% FITC‐labelled PEG (green) and B) 1 w% RITC‐labelled polymer (magenta), and C) <1 wt.% RITC‐labelled Myoglobin (magenta). D) A diagram showing the additional compounds (green) preferentially located in the aqueous phase, and the block copolymer coacervates (blue and red). Green scale bar = 20 µm.

The effect of adding FITC‐labelled poly(ethylene glycol) (PEG),^[^ ^42^ ^]^ and RITC‐labelled Myoglobin^[^ ^43^ ^]^ on DHBC coacervate formation was also investigated by adding varying concentrations of the two compounds to 25 wt.% POEGMA_9k_‐*b‐*POEtOx_8k_ aqueous solutions. At concentrations below 1 wt.%, both Myoglobin and PEG preferentially localised in the aqueous continuous phase rather than the polymer droplet phase (**Figure** **4**). This behaviour could be beneficial for potential applications in catalysis.

**Figure 5:**
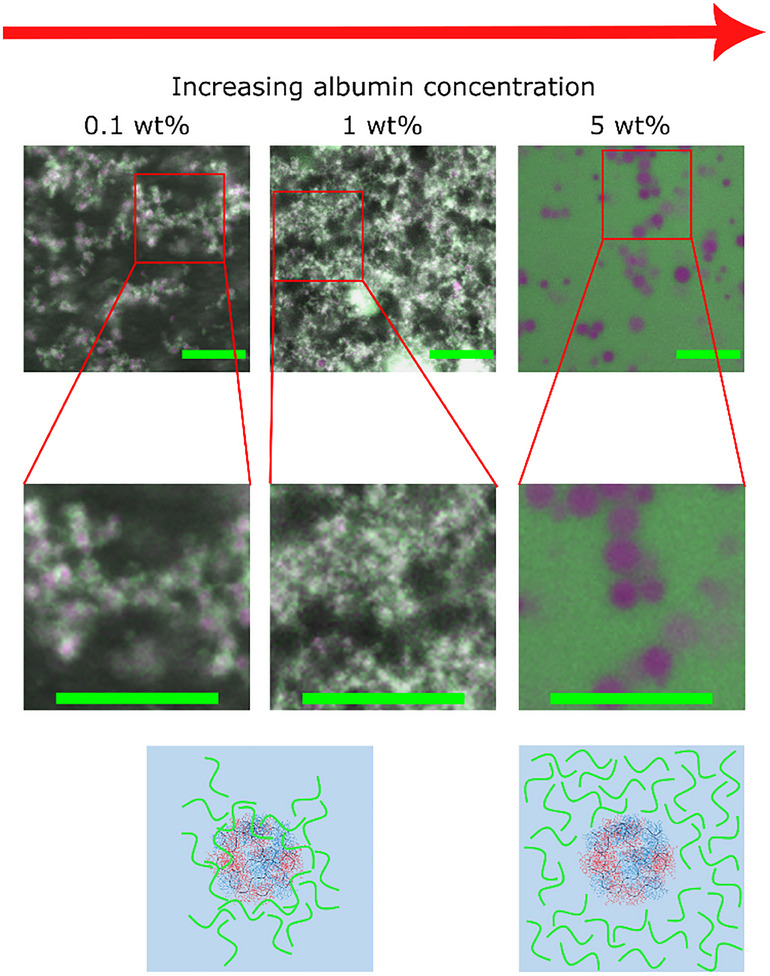
CLSM images of 25 wt.% aqueous solutions of RITC‐labelled POEGMA_9k_‐*b‐*POEtOx_8k_ (magenta), and FITC‐labelled BSA at increasing concentrations (green) including additional magnification. Representations of the location of BSA in solution (green). Green scale bar = 20 µm.

Finally, the effect of adding bovine serum albumin (BSA) was investigated using a 25 wt.% aqueous solution of POEGMA_9k_‐*b‐*POEtOx_8k_ block copolymer (**Figure** **5**). At an initial concentration of 0.1 wt.%, BSA showed no preference for either phase and instead localized at the interface between the polymer droplet phase and the aqueous continuous phase. This effect persisted at an increased concentration of 1 wt.%, with the polymer droplets appearing smaller compared to those observed at 0.1 wt.% BSA. Previous studies have explored the accumulation of BSA at water–water interfaces,^[^ ^44^ ^]^ where it has been used to stabilize the interface of w/w emulsions, such as those involving PEG and DEX.^[^ ^45^ ^]^ The surface activity of BSA has been leveraged in various ATPS‐based technologies, for example, in membrane formation with nanospheres,^[^ ^46^ ^]^ or as a stabilizer in BSA‐loaded polymer particles in w/w emulsions for biocatalysis.^[^ ^47^ ^]^ At a concentration of 5 wt.%, BSA was found to be enriched in the aqueous continuous phase, further resembling a traditional ATPS‐derived w/w emulsion. Based on the results with BSA and DEX, we hypothesize that DHBCs may behave similarly to homopolymers in common ATPSs, i.e. polymer segregation is observed by preferential partitioning into opposite phases.

## 3 Conclusion

In this study, we demonstrate the formation of simple coacervate droplets using a double hydrophilic brush block copolymer without the use of external triggers or charged polymers, combining POEGMA and POEtOx. At a high concentration of 25 wt.%, the DHBC forms coacervate droplets with sizes in the low micrometre range, which exhibit relatively high stability over an extended period, with only minor coalescence observed after 4 weeks. No coacervation was observed at low concentrations. Furthermore, copolymers of the two individual macromonomers failed to show coacervation, highlighting that the DHBC architecture is crucial for the formation of the desired structures. The addition of guest polymers and biomacromolecules at low concentrations revealed specific partitioning behaviour, with a preference for either the polymer or the aqueous phase. At higher guest concentrations, enrichment in the aqueous phase was observed, consistent with common w/w emulsions, although in this case, no stabilizers were employed. These findings open a new direction for coacervate systems with potential applications in biotechnology, synthetic cell environment mimics, and drug delivery.

## Conflict of Interest

The authors declare no conflict of interest.

## Supporting information



Supporting Information

## Data Availability

The data that support the findings of this study are available from the corresponding author upon reasonable request.
