# Supplementary material for: Self‐Coacervation of Oligo(ethylene glycol) and Oligo(2‐ethyl‐2‐oxazoline)‐Based Double Hydrophilic Brush Block Copolymers in Aqueous Solution
Source: Macromol Rapid Commun. 2025 Apr 18;46(23):2500151. doi: 10.1002/marc.202500151 (PMC12687681; doi:10.1002/marc.202500151)
Supplement: Supplementary file 1 — Supporting Information [file MARC-46-2500151-s001.pdf]

Supporting Information

Self-coacervation of oligo(ethylene glycol) and oligo(2-ethyl-2-oxazoline)-based double hydrophilic brush block copolymers in aqueous solution

*Niamh Bayliss, Matilde Concilio, Alexander Plucinski, Gokhan Yilmaz, C. Remzi Becer**
*Bernhard V. K. J. Schmidt**

N. Bayliss, A. Plucinski, B. V. K. J. Schmidt
School of Chemistry, University of Glasgow, G128QQ, UK
E-mail: Bernhard.schmidt@glasgow.ac.uk

M. Concilio, G. Yilmaz, C. R. Becer
Department of Chemistry, University of Warwick, Coventry, CV4 7AL, UK
E-mail: Remzi.Becer@warwick.ac.uk

Experimental Section

Materials

Azobisisobutyronitrile (AIBN, 99%, Sigma Aldrich, recrystallized from methanol), Bovine Serum Albumin – fluorescein isothiocyanate (FITC-BSA, Sigma Aldrich), chloroform (>99.0%, $CHCl_3$, Sigma Aldrich), 4-cyano-4-(phenylcarbonothioylthio)pentanoic acid (CPPA, Sigma Aldrich), dextran (DEX, TCI, 40 kg mol^{-1}), dioxane (99%, Sigma-Aldrich, passed over a column of basic aluminium oxide), *N,N*-diisopropylethylamine (99.5%, DIPEA, Sigma Aldrich), *N,N*-dimethylformamide (99%, Sigma Aldrich), dimethylsulfoxide (DMSO, dry over molecular sieves, Acros), Fluorescein isothiocyanate (FITC, Sigma-Aldrich), Fluorescein isothiocyanate dextran (FITC-DEX, Sigma Aldrich), hexylamine (99%, Sigma Aldrich), anhydrous magnesium sulfate (≥99.5%, Sigma Aldrich), methacrylic acid (99%, Sima Aldrich), Myoglobin from equine skeletal muscle (Mb, Sigma Aldrich), oligo(ethylene glycol) methyl ether methacrylate (OEGMA, M_n=950 g/mol, Sigma-Aldrich, dissolved in THF, passed over a column of basic aluminium oxide and precipitated in diethyl ether), poly(ethylene glycol) (PEG, Sigma Aldrich, 35 kg mol^{-1}), Rhodamine B isothiocyanate (RITC, Sigma-Aldrich), tetrahydrofuran (THF, extra dry, Acros Organics) were used as received unless otherwise noted. 2-Ethyl-2-oxazoline (99+%, Acros Organics, EtOx) was dried over calcium hydride and distilled under reduced pressure prior to use. Methyl tosylate (98%, Aldrich, MeTos) was distilled under reduced pressure and stored under nitrogen. Extra dry acetonitrile (99.9+%, CH_3CN) was purchased from Fisher Scientific and stored over molecular sieves and under inert atmosphere. Deionized water was obtained from a Sartorius Arium pro ultrapure water system. FITC-labelled PEG[1] and RITC-labelled Myoglobin[2] were synthesized according to the literature.

Analytical methods

^{1}H-NMR and DOSY spectra were recorded in deuterium oxide (D_2O, Aldrich) or d-DMSO (Sigma Aldrich) at ambient temperature at 400 MHz with a Bruker Ascend400. Conversion was determined from the ^{1}H NMR spectra using the polymer peak at 3.30 ppm and the macromonomer peak at 6.0 ppm for POEGMA and the polymer peak at 1.0 ppm and the macromonomer peak at 6.1 ppm for POEtOx. Block ratios were determined by comparing the terminal methyl signals for the POEGMA and POETOX blocks around 3.30 and 2.95 ppm.

All GPC data was collected using an Agilent Infinity 1260 instrument equipped with differential refractive index (DRI) detector. The system included two Agilent Polymer Labs (PL) aquagel-OH Mixed M columns (300 × 7.5 mm) and a PL aquagel-OH Guard column. The mobile phase

consisted of 0.1 M $NaNO_3$ (aq.) and MeOH (80:20% v/v), running at a flow rate of 1 mL/min at 25°C. A third-order conventional calibration was established using Agilent polyethylene glycol/oxide (PEG/PEO) EasiVials, with DRI data covering a molecular weight range of 106 and 427,500 g/mol. All sample analyses were performed using Agilent GPC/SEC Software. All samples were passed through 0.2 μm Nylon filter before analysis.

Confocal laser scanning microscopy (CLSM) and bright field microscopy were performed on Zeiss LSM710 confocal microscope (Zeiss, Göttingen, Germany) and software Carl Zeiss ZEN 2011 v7.0.3.286. LD EC Epiplan NEUFLUAR 50X, 0.55 DIC (Carl Zeiss, White Plains, NY, USA), NEUFLUAR 20X, 0.55 DIC (Carl Zeiss, White Plains, NY, USA) and N-Achroplan 10x/0.25 Ph 1 (Carl Zeiss, White Plains, NY, USA) objectives were used. All samples were prepared in a CELLview (Greiner Bio-One, Stonehouse, UK) 35 mm plastic cell culture dish with a borosilicate glass bottom. The images were taken with three different channels, two for the particular dyes (RITC or FITC) and one for a bright field image. The size of the coacervate droplets was determined over 30 particles from bright field images and averaged. The error is based on the standard deviation.

OEtOx synthesis

According to the literature,[3] EtOx (4.98 g, 50.2 mmol, 10 eq.) and CH_3CN (12.5 mL) were transferred into a round bottom flask equipped with a magnetic stirring bar. The final monomer concentration was 4.0 M. The reaction mixture was degassed with a nitrogen flow for 30 minutes before adding MeTos (0.76 mL, 5.1 mmol, 1 eq.). The ratio of [monomers]:[I] was 25:1. The reaction mixture was heated up to 100 °C for 50 min. In the meantime, a solution containing methacrylic acid (1.3 mL, 15.1 mmol, 3 eq.) and DIPEA (2.6 mL, 15.1 mmol, 3 eq.) in CH_3CN (3 mL) was prepared and degassed with a nitrogen flow for 30 min. After completion of the first reaction, the MAA/DIPEA solution was directly added to the flask containing the living oxazoline chains, and the reaction mixture was reacted at 80 °C overnight. Afterwards, the solvent was evaporated, and the polymer was redissolved in chloroform. This solution was washed with saturated aqueous sodium hydrogen carbonate (x2) and saturated brine (x2), dried with anhydrous magnesium sulfate and filtered. The solvent was evaporated under reduced pressure and the resulting white polymer was dried under vacuum and stored at -18 °C.

RAFT-polymerization of $OEGMA_{5k}$

Destabilized OEGMA (1.0 g, 1 mmol, 7 eq.), CPPA (39.4 mg, 0.14 mmol, 1.0 eq.), AIBN (4.8 mg, 0.02 mmol, 0.2 eq.) and DMF (3 mL) were mixed in a round bottom flask containing

a stirring bar and sealed with a septum. The solution was bubbled for 30 min with nitrogen and the polymerization was initiated by heating to 65 °C in an oil bath. The polymerization was stopped after 24 h (67% conversion). Subsequently, the polymer was dialysed against deionized water (Spectra/Por 3500 Da) for 3 days. Finally, the sample was freeze-dried and an off-red solid (896 mg, 3700 g/mol) was obtained.

RAFT-polymerization of $OEGMA_{9k}$

Destabilized OEGMA (1.0 g, 1 mmol, 10 eq.), CPPA (27.9 mg, 0.1 mmol, 1.0 eq.), AIBN (3.3 mg, 0.02 mmol, 0.2 eq.), deionized water (0.6 mL) and dioxane (2.4 mL) were mixed in a round bottom flask containing a stirring bar and sealed with a septum. The solution was bubbled for 30 min with nitrogen and the polymerization was initiated by heating to 65 °C in an oil bath. The polymerization was stopped after 24 h (89% conversion). Subsequently, the polymer was dialysed against deionized water (Spectra/Por 3500 Da) for 3 days. Finally, the sample was freeze-dried and an off-red solid (846 mg, 7200 g/mol) was obtained.

RAFT-polymerization of $OEGMA_{13k}$

Destabilized OEGMA (2.0 g, 2 mmol, 28.6 eq.), CPPA (19.6 mg, 0.07 mmol, 1.0 eq.), AIBN (2.4 mg, 0.015 mmol, 0.2 eq.) and DMF (6 mL) were mixed in a round bottom flask containing a stirring bar and sealed with a septum. The solution was bubbled for 30 min with nitrogen and the polymerization was initiated by heating to 65 °C in an oil bath. The polymerization was stopped after 24 h (64% conversion). Subsequently, the polymer was dialyzed against deionized water (Spectra/Por 3500 Da) for 3 days. Finally, the sample was freeze-dried and an off-red solid (977 mg, 7000 g/mol) was obtained.

Block copolymer formation for $POEGMA_{5k}$-*b*-$POEtOx_{4k}$

$POEGMA_{5k}$ (250 mg, 0.05 mmol, 1 eq.), OEtOx (250 mg, 0.25 mmol, 5 eq.), AIBN (1.6 mg, 10 µmol, 0.2 eq.), deionized water (0.3 mL) and dioxane (1.2 mL) were mixed in a round bottom flask containing a stirring bar and sealed with a septum. The solution was bubbled for 30 min with nitrogen and the polymerization was initiated by heating to 65 °C in an oil bath. The polymerization was stopped after 24 h (76% conversion). Subsequently, the polymer was dialyzed against deionized water (Spectra/Por 3500 Da) for 3 days. Finally, the sample was freeze-dried and an off-red solid (390 mg, M_n= 7300 g/mol) was obtained.

Block copolymer formation for $POEGMA_{7k}$-b-$POEtOx_{8k}$

$POEGMA_{7k}$ (250 mg, 0.025 mmol, 1 eq.), OEtOx (250 mg, 0.25 mmol, 10 eq.), AIBN (0.8 mg, 5 µmol, 0.2 eq.) deionized water (0.3 mL) and dioxane (1.2 mL) were mixed in a round bottom flask containing a stirring bar and sealed with a septum. The solution was bubbled for 30 min with nitrogen and the polymerization was initiated by heating to 65 °C in an oil bath. The polymerization was stopped after 24 h (80% conversion). Subsequently, the polymer was dialyzed against deionized water (Spectra/Por 10000 Da) for 3 days. Finally, the sample was freeze-dried and an off-red solid (346 mg, M_n= 8600 g/mol) was obtained.

Block copolymer formation for $POEGMA_{13k}$-*b*-$POEtOx_{9k}$

$POEGMA_{13k}$ (250 mg, 0.0125 mmol, 1 eq.), OEtOx (250 mg, 0.25 mmol, 20 eq.), AIBN (0.4 mg, 2.5 µmol, 0.2 eq.), deionized water (0.3 mL) and dioxane (1.2 mL) were mixed in a round bottom flask containing a stirring bar and sealed with a septum. The solution was bubbled for 30 min with nitrogen and the polymerization was initiated by heating to 65 °C in an oil bath. The polymerization was stopped after 24 h (77% conversion). Subsequently, the polymer was dialyzed against deionized water (Spectra/Por 10000 Da) for 3 days. Finally, the sample was freeze-dried and an off-red solid (326 mg, M_n= 12300 g/mol) was obtained.

Exemplary fluorescent labelling of block copolymer RITC-$POEGMA_{7k}$-b-$POEtOx_{2k}$

In a dry nitrogen purged Schlenk tube, *$POEGMA_{7k}$-b*-$POEtOx_{2k}$ (50 mg, 0.0025 mmol, 1.0 eq.) was dissolved in dry DMSO (2.5 mL). Hexylamine (0.8 µL, 0.006 mmol, 2.4 eq.) was added and the solution stirred for 24 h at 50 °C. After cooling to ambient temperature, RITC (10.1 mg, 0.019 mmol, 7.5 eq.) was added under nitrogen flow and the mixture stirred again for 24 h at 50 °C. Subsequently, the polymer was dialyzed against deionized water (Spectra/Por 3500 Da) for 3 days. Finally, the sample was freeze-dried and a red solid (49 mg) was obtained.

RAFT copolymerization of OEGMA and OEtOx for P($OEGMA_{0.8}$-*co*-$OEtOx_{0.2}$)

Destabilized OEGMA (0.8 g, 0.8 mmol, 20 eq.), OEtOx (0.2 g, 0.2 mmol, 5 eq.), CPPA (9.8 mg, 0.04 mmol, 1.0 eq.), AIBN (1.2 mg, 7 µmol, 0.2 eq.) and DMF (3 mL) were mixed in a vial (7 mL) containing a stirring bar and sealed with a septum. The solution was bubbled for 30 min with nitrogen and the polymerization was initiated by heating to 65 °C in an oil bath. The polymerization was stopped after 24 h (79% conversion for OEGMA, 50% conversion for OEtOx). Subsequently, the polymer was dialyzed against deionized water (Spectra/Por 3500

Da) for 3 days. Finally, the sample was freeze-dried and an off-red solid (812 mg, M_n= 17600 g/mol) was obtained.

RAFT copolymerization of OEGMA and OEtOx for P(OEGMA$_{0.5}$-*co*-OEtOx$_{0.5}$)

Destabilized OEGMA (0.5 g, 0.5 mmol, 12.5 eq.), OEtOx (0.5 g, 0.5 mmol, 12.5 eq.), CPPA (9.8 mg, 0.04 mmol, 1.0 eq.), AIBN (1.2 mg, 7 µmol, 0.2 eq.) and DMF (3 mL) were mixed in a vial (7 mL) containing a stirring bar and sealed with a septum. The solution was bubbled for 30 min with nitrogen and the polymerization was initiated by heating to 65 °C in an oil bath. The polymerization was stopped after 24 h (96% conversion for OEGMA, 66% conversion for OEtOx). Subsequently, the polymer was dialyzed against deionized water (Spectra/Por 3500 Da) for 3 days. Finally, the sample was freeze-dried and an off-red solid (685 mg, M_n= 20100 g/mol) was obtained.

RAFT copolymerization of OEGMA and OEtOx for P(OEGMA$_{0.2}$-*co*-OEtOx$_{0.8}$)

Destabilized OEGMA (0.2 g, 0.2 mmol, 4 eq.), OEtOx (0.8 g, 0.8 mmol, 16 eq.), CPPA (13.6 mg, 0.05 mmol, 1.0 eq.), AIBN (1.6 mg, 10 µmol, 0.2 eq.), deionized water (0.6 mL) and dioxane (2.4 mL) were mixed in a vial (7 mL) containing a stirring bar and sealed with a septum. The solution was bubbled for 30 min with nitrogen and the polymerization was initiated by heating to 65 °C in an oil bath. The polymerization was stopped after 24 h (quantitative conversion for OEGMA, 82% conversion for OEtOx). Subsequently, the polymer was dialyzed against deionized water (Spectra/Por 3500 Da) for 3 days. Finally, the sample was freeze-dried and an off-red solid (769 mg, M_n= 7400 g/mol) was obtained.

Exemplary fluorescent labelling of copolymer P(OEGMA$_{0.5}$-co-OEtOx$_{0.5}$)

In a dry nitrogen purged Schlenk tube, POEGMA$_{0.5}$-*co*-POEtOx$_{0.5}$ (50 mg, 0.005 mmol, 1.0 eq.) was dissolved in dry DMSO (2.5 mL). Hexylamine (1.6 µL, 0.0125 mmol, 2.5 eq.) was added and the solution stirred for 24 h at 50 °C. After cooling to ambient temperature, FITC (14.5 mg, 0.038 mmol, 7.5 eq.) was added under nitrogen flow and the mixture stirred again for 24 h at 50 °C. Subsequently, the polymer was dialysed against deionized water (Spectra/Por 3500 Da) for 3 days. Finally, the sample was freeze-dried and a yellow solid (47 mg) was obtained.

Preparation of block copolymer CLSM samples

In order to visualize the aggregation of the POEGMA-*b*-POEtOx block copolymer samples of $POEGMA_{4k}$-*b*-$POEtOx_{4k}$, $POEGMA_{7k}$-*b*-$POEtOx_{1k}$ and $POEGMA_{7k(a)}$-*b*-$POEtOx_{5k}$ were dissolved in both 25 wt% and 15 wt% aqueous solutions, with 1 wt% of the polymer sample being RITC labelled. The samples were then agitated before CLSM analysis. A similar procedure was used for copolymer reference samples.

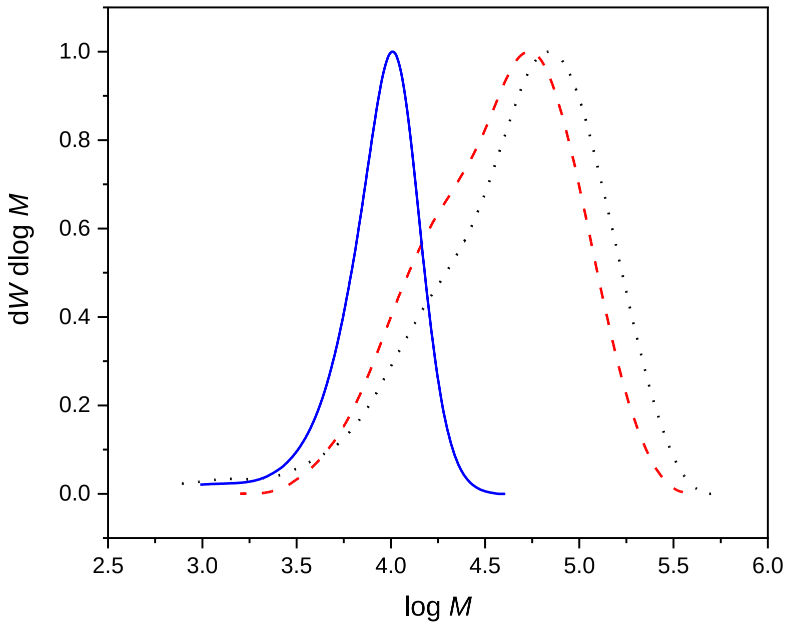

Figure S1. Molecular weight distributions of copolymers P($OEGMA_{0.8}$-*co*-$OEtOx_{0.2}$) (black dotted line), P($OEGMA_{0.5}$-*co*-$OEtOx_{0.5}$) (red dashed line) and P($OEGMA_{0.2}$-*co*-$OEtOx_{0.8}$) (blue solid line) obtained in 0.1 M $NaNO_3$ (aq.) and MeOH (80:20% v/v) with PEG calibration.

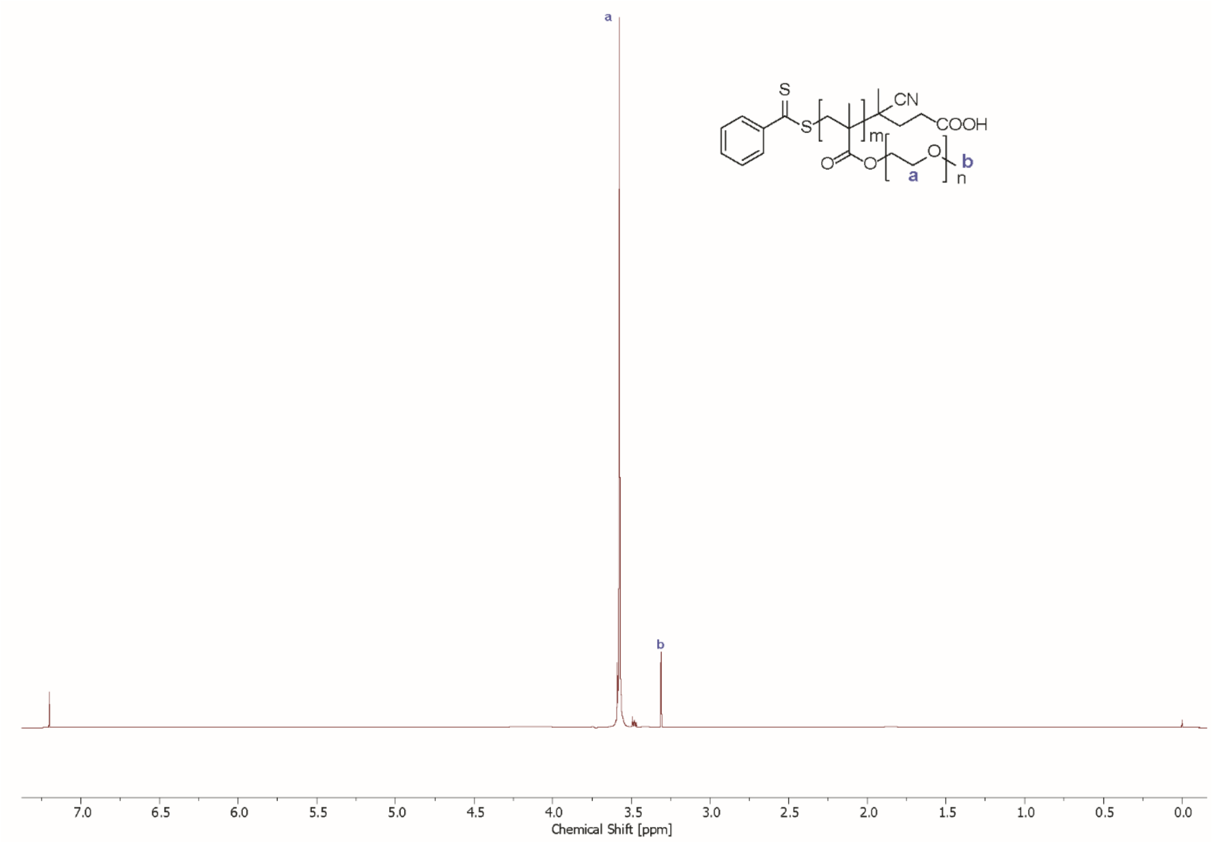

Figure S2. ^{1}H NMR of $POEGMA_{5k}$ measured in $CDCl_3$.

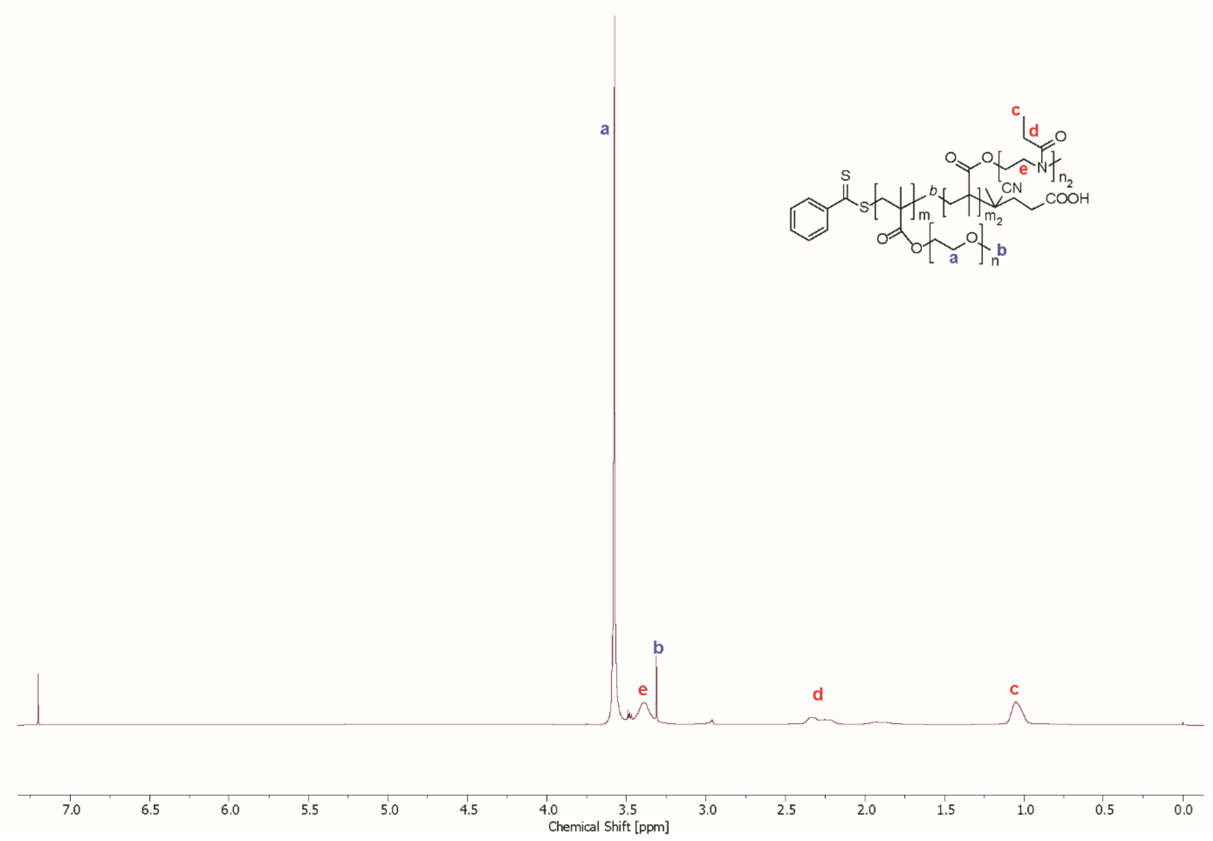

Figure S3. ^{1}H NMR of $POEGMA_{5k}$-*b*-$POEtOx_{4k}$ measured in $CDCl_3$.

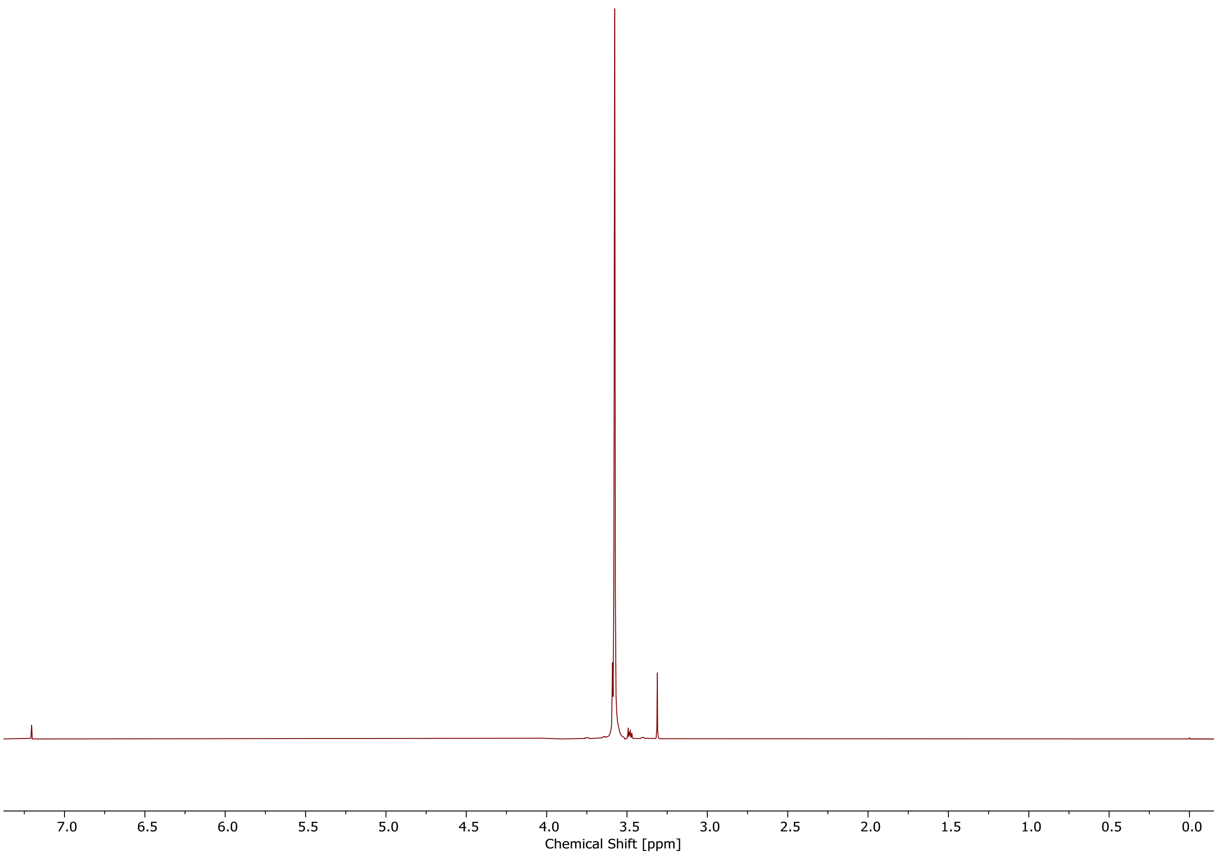

Figure S4. ^{1}H NMR of POEGMA$_{9k}$ measured in $CDCl_3$.

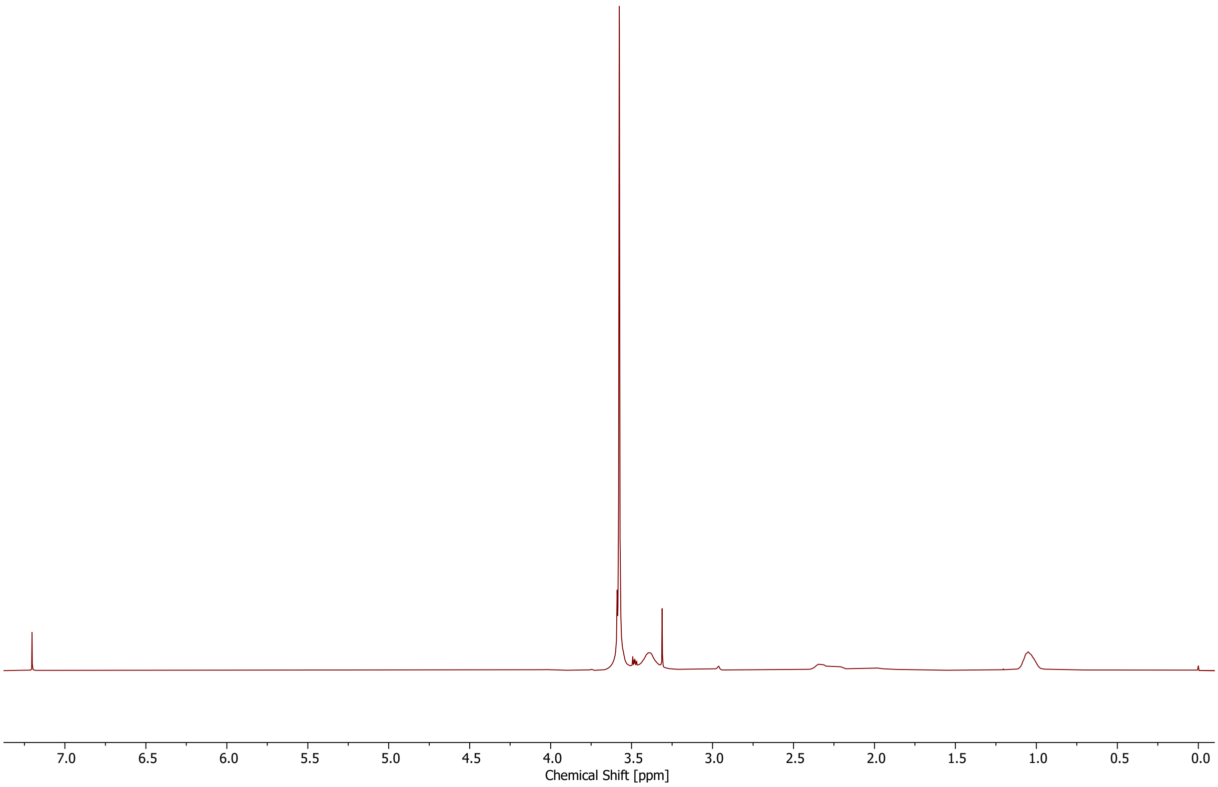

Figure S5. ^{1}H NMR of POEGMA$_{9k}$-*b*-POEtOx$_{8k}$ measured in $CDCl_3$.

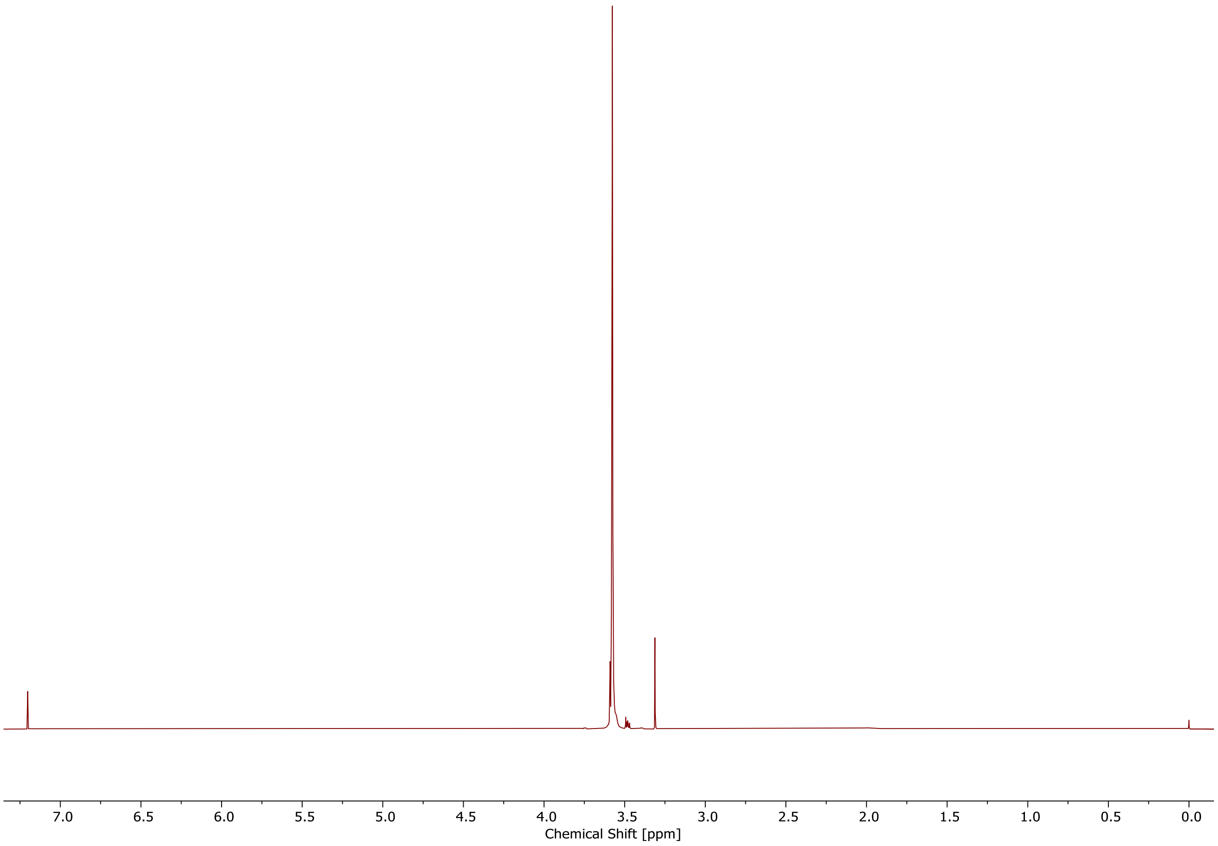

Figure S6. ^{1}H NMR of $POEGMA_{13k}$ measured in $CDCl_3$.

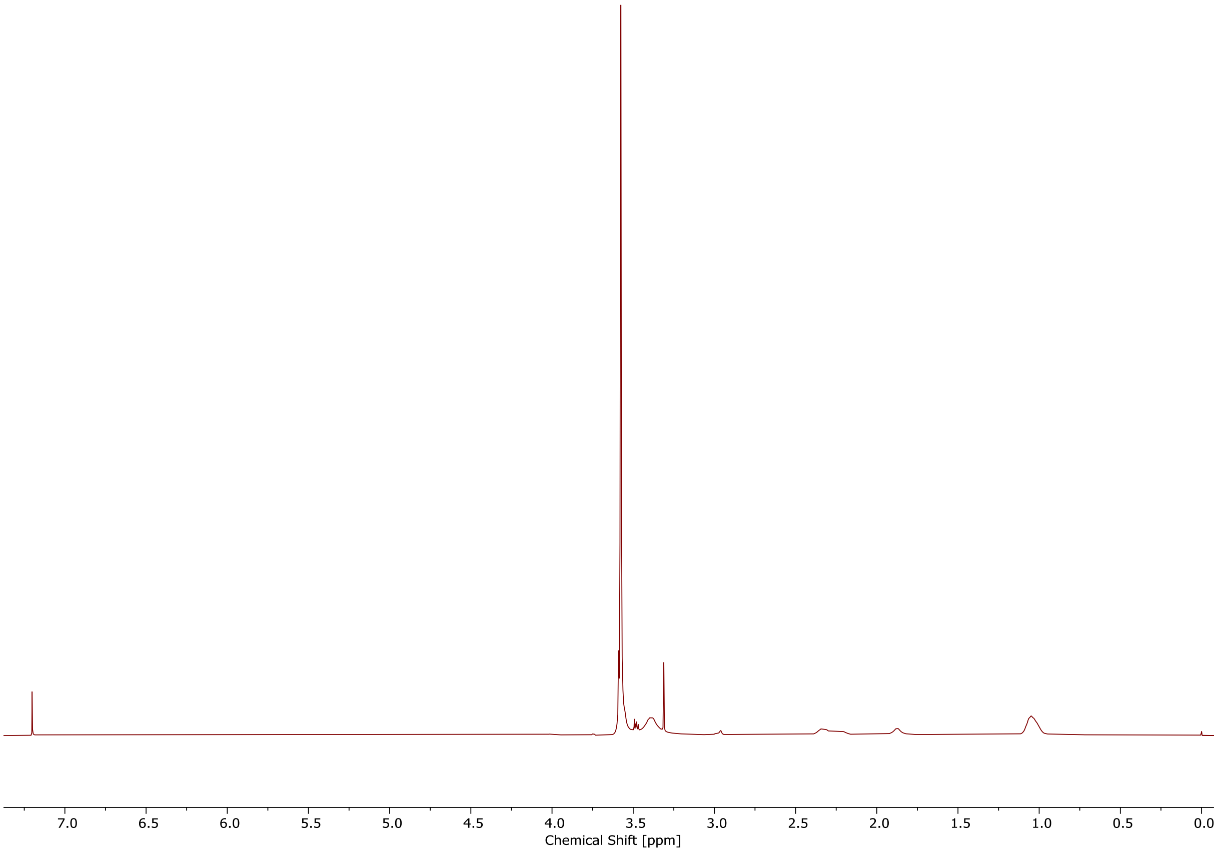

Figure S7. ^{1}H NMR of $POEGMA_{13k}$-*b*-$POEtOx_{9k}$ measured in $CDCl_3$.

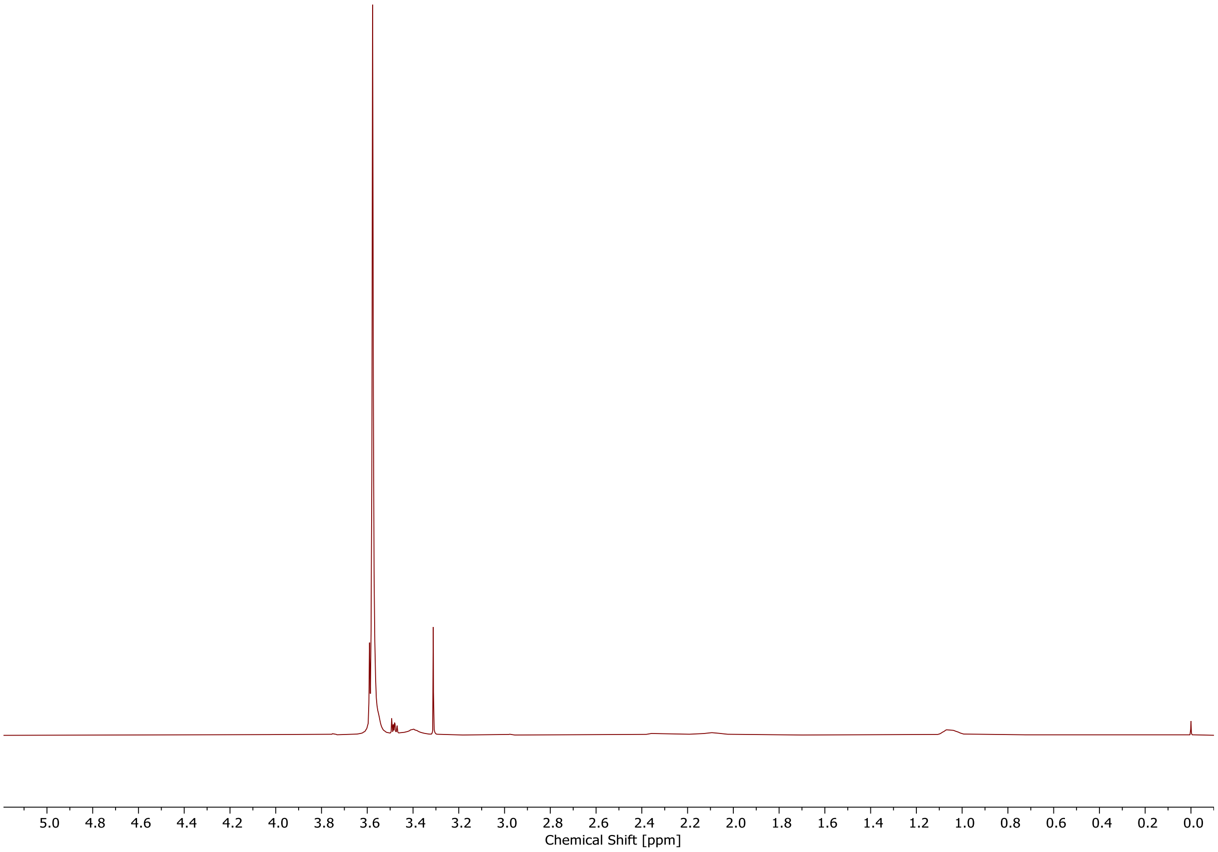

Figure S8. ^{1}H NMR of P(OEGMA$_{0.8}$-*co*-OEtOx$_{0.2}$) measured in $CDCl_3$.

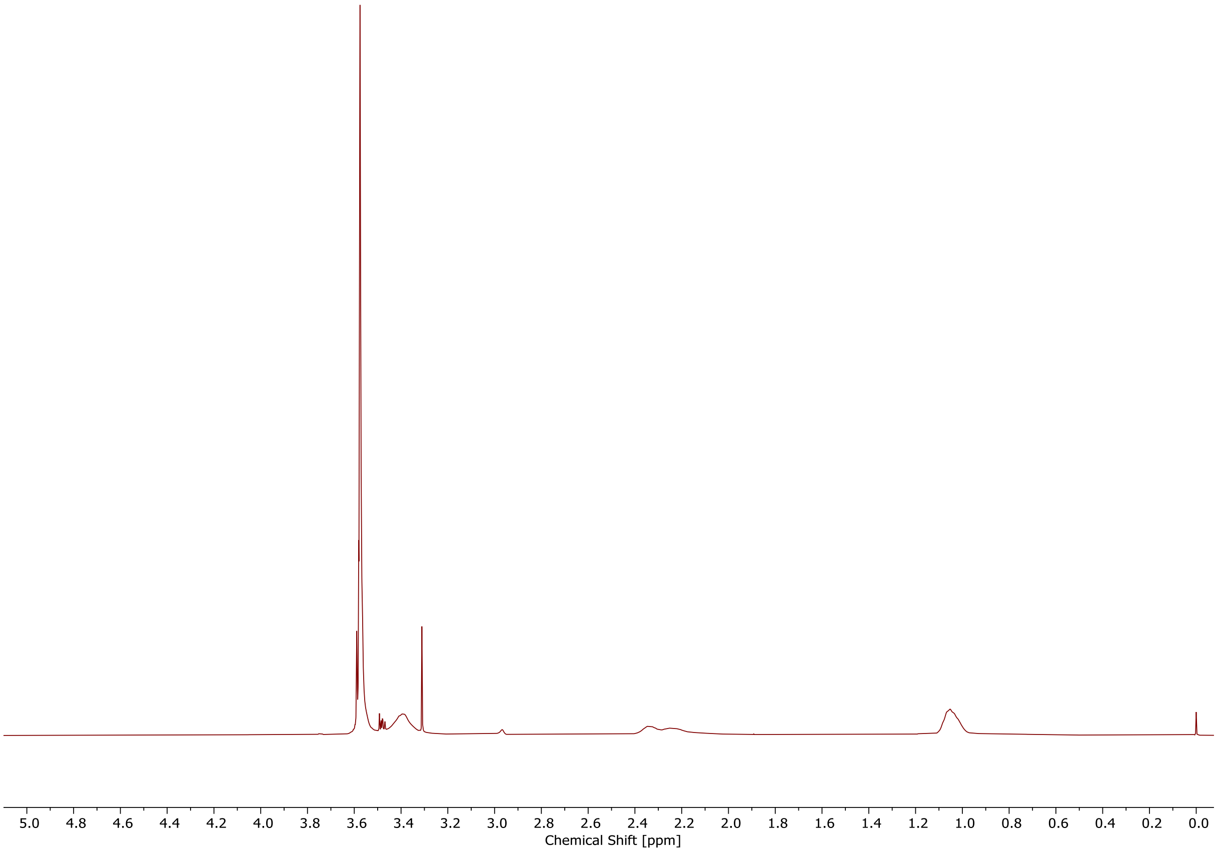

Figure S9. ^{1}H NMR of P(OEGMA$_{0.5}$-*co*-OEtOx$_{0.5}$) measured in $CDCl_3$.

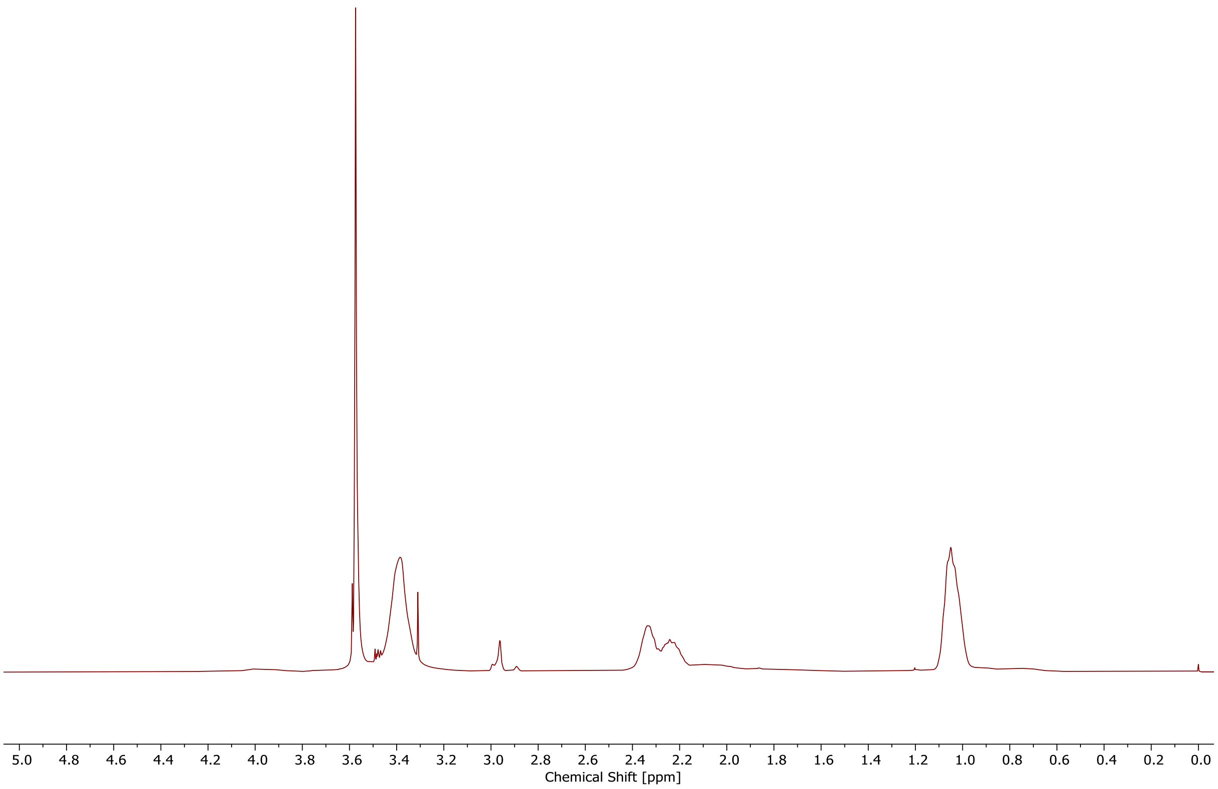

Figure S10. ^{1}H NMR of P(OEGMA$_{0.2}$-*co*-OEtOx$_{0.8}$) measured in $CDCl_3$.

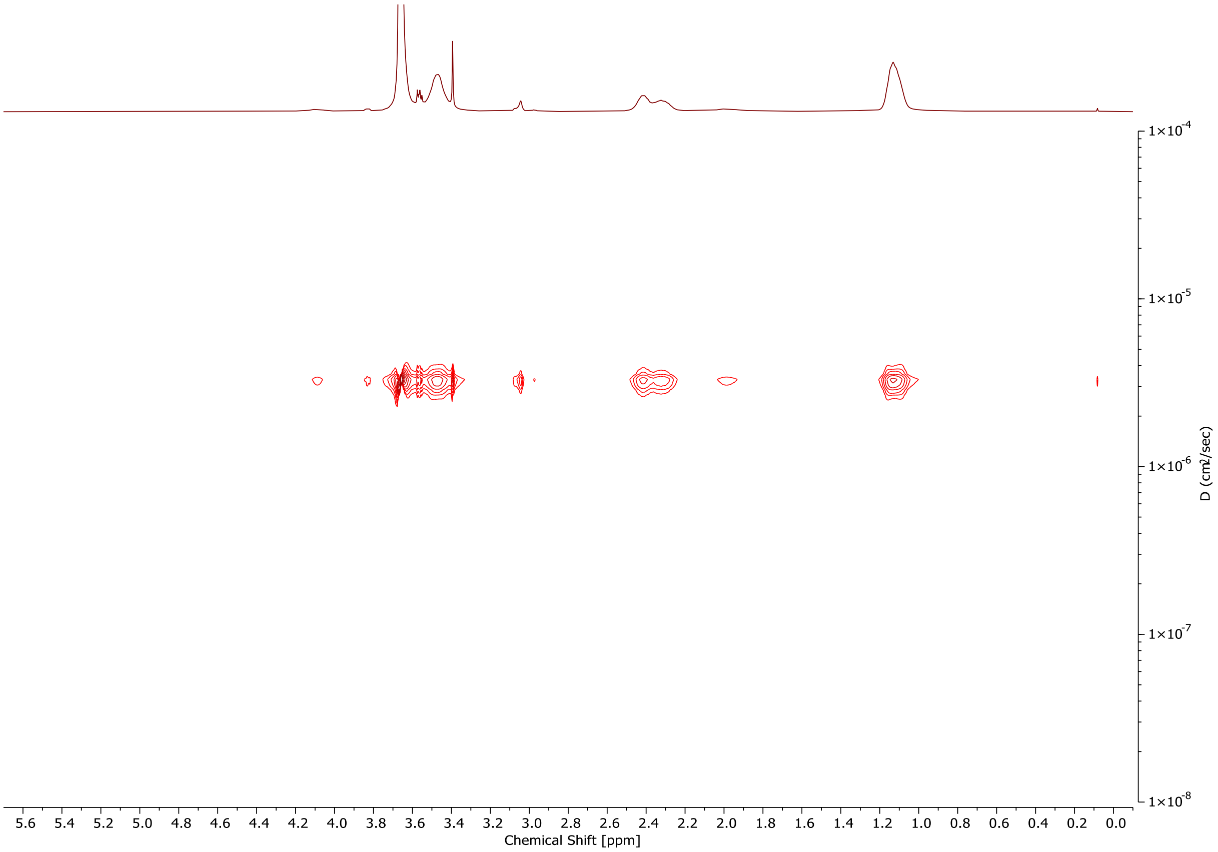

Figure S11. DOSY of POEGMA$_{5k}$-*b*-POEtOx$_{4k}$ measured in $CDCl_3$.

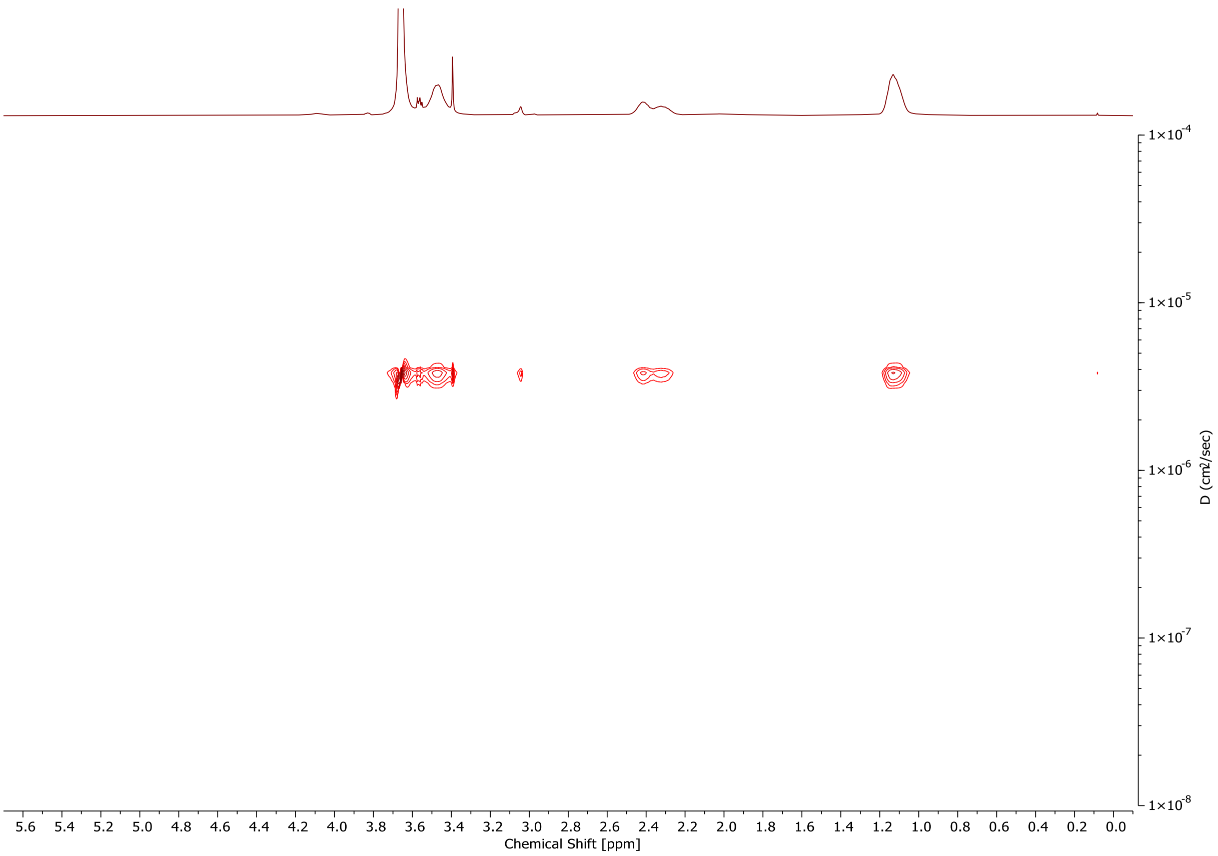

Figure S12. DOSY of POEGMA$_{9k}$-*b*-POEtOx$_{8k}$ measured in $CDCl_3$.

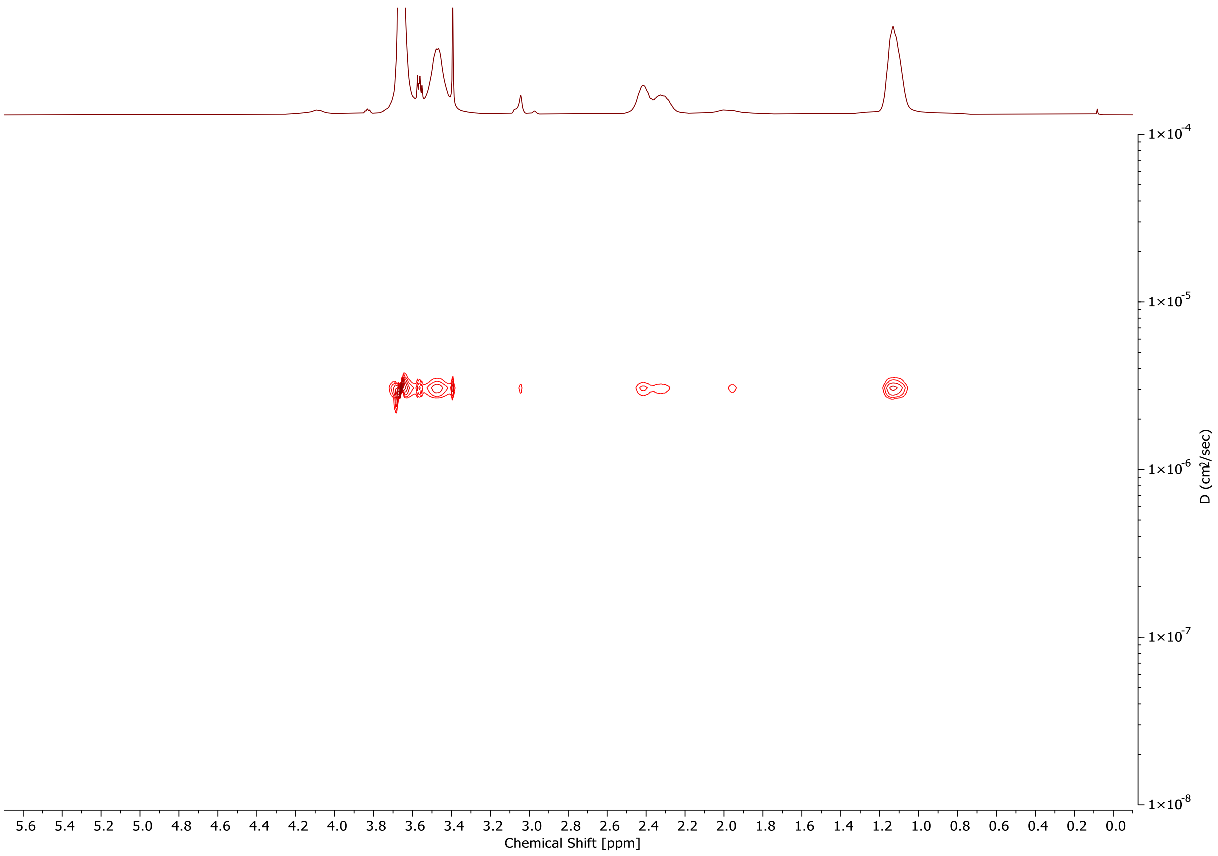

Figure S13. DOSY of POEGMA$_{13k}$-*b*-POEtOx$_{9k}$ measured in $CDCl_3$.

Table S1. Coacervate droplet sizes at a polymer concentration of 25 wt% after 24 h and 4 weeks.

Polymer	*d* [µm] after 24 h[a]	*d* [µm] after 4 weeks[a]	Increment factor
POEGMA$_{5k}$-*b*-POEtOx$_{4k}$	2.50 ± 1.0	7.98 ± 3.5	3.2
POEGMA$_{9k}$-*b*-POEtOx$_{8k}$	1.64 ± 0.6	2.86 ± 0.92	1.7
POEGMA$_{13k}$-*b*-POEtOx$_{9k}$	1.37 ± 0.7	1.90 ± 0.82	1.4

[a)] Obtained from CLSM images and averaged from 100 droplets

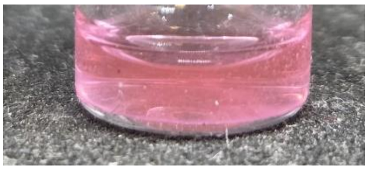

Figure S14. 25 wt% POEGMA$_{5k}$-*b*-POEtOx$_{4k}$ in deionized water (1 wt% of the respective RITC labelled POEGMA-*b*-POEtOx added) solution after 4 weeks.

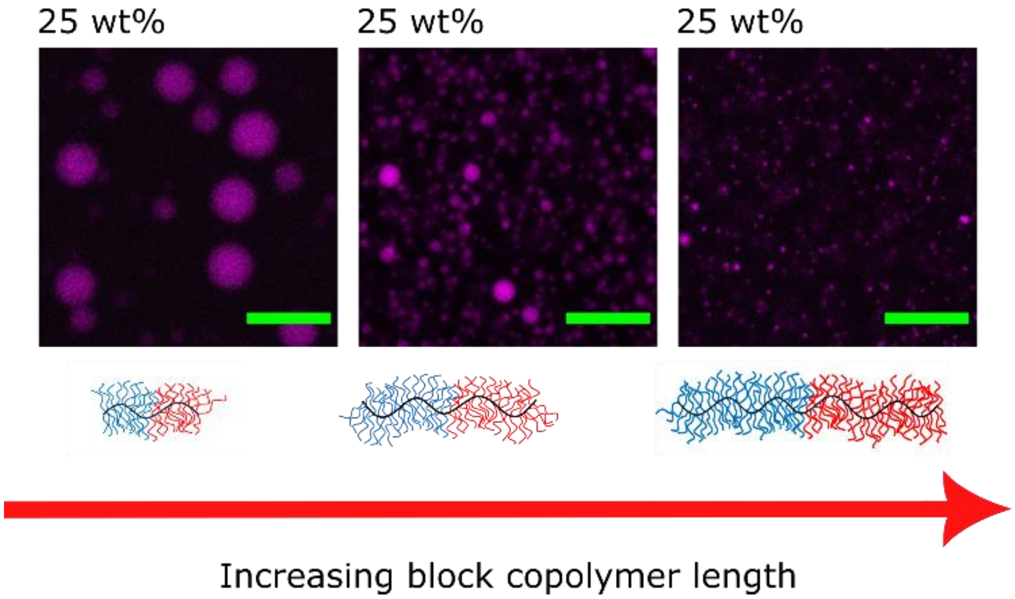

Figure S15. 25 wt% POEGMA$_{5k}$-*b*-POEtOx$_{4k}$, POEGMA$_{9k}$-*b*-POEtOx$_{8k}$ and POEGMA$_{13k}$-*b*-POEtOx$_{9k}$ in deionized water (1 wt% of the respective RITC labelled POEGMA-*b*-POEtOx added) shown in magenta with varying molecular weight imaged after 4 weeks. Green scale bar = 20 µm.

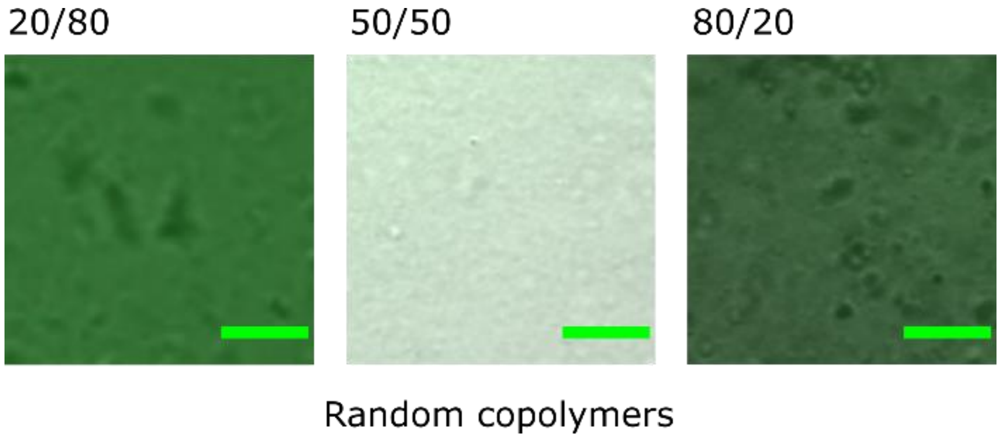

Figure S16. 25 wt% random copolymers in deionized water (1 wt% of respective FITC labelled P(OEGMA-*co*-OEtOx) added) shown in green with varying ratios P($OEGMA_{0.2}$-*co*-$OEtOx_{0.8}$)(20/80), P($OEGMA_{0.5}$-*co*-$OEtOx_{0.5}$)(50/50) and P($OEGMA_{0.8}$-*co*-$OEtOx_{0.2}$)(80/20). Green scale bar = 20 µm.

References

[1] J.-B. Masclef, E. M. N. Acs, J. Koehnke, J. Prunet, B. V. K. J. Schmidt, *Macromolecules* **2024**, *57*, 6013.
[2] N. Martin, J.-P. Douliez, Y. Qiao, R. Booth, M. Li, S. Mann, *Nat. Commun.* **2018**, *9*, 3652.
[3] M. Concilio, N. Nguyen, S. C. L. Hall, S. Huband, C. R. Becer, *Macromolecules* **2023**, *56*, 7961.
